# Artificial intelligence-powered innovations in periodontal diagnosis: a new era in dental healthcare

**DOI:** 10.3389/fmedt.2024.1469852

**Published:** 2025-01-10

**Authors:** Jarupat Jundaeng, Rapeeporn Chamchong, Choosak Nithikathkul

**Affiliations:** ^1^ Ph.D. in Health Science Program, Faculty of Medicine, Mahasarakham University, Mahasarakham, Thailand; ^2^ Tropical Health Innovation Research Unit, Faculty of Medicine, Mahasarakham University, Mahasarakham, Thailand; ^3^ Dental Department, Fang Hospital, Chiang Mai, Thailand; ^4^ Department of Computer Science, Faculty of Informatics, Mahasarakham University, Mahasarakham, Thailand

**Keywords:** artificial intelligence, periodontal disease, periodontitis diagnosis, panoramic radiographs, convolutional neural networks (CNNs)

## Abstract

**Background:**

The aging population is increasingly affected by periodontal disease, a condition often overlooked due to its asymptomatic nature. Despite its silent onset, periodontitis is linked to various systemic conditions, contributing to severe complications and a reduced quality of life. With over a billion people globally affected, periodontal diseases present a significant public health challenge. Current diagnostic methods, including clinical exams and radiographs, have limitations, emphasizing the need for more accurate detection methods. This study aims to develop AI-driven models to enhance diagnostic precision and consistency in detecting periodontal disease.

**Methods:**

We analyzed 2,000 panoramic radiographs using image processing techniques. The YOLOv8 model segmented teeth, identified the cemento-enamel junction (CEJ), and quantified alveolar bone loss to assess stages of periodontitis.

**Results:**

The teeth segmentation model achieved an accuracy of 97%, while the CEJ and alveolar bone segmentation models reached 98%. The AI system demonstrated outstanding performance, with 94.4% accuracy and perfect sensitivity (100%), surpassing periodontists who achieved 91.1% accuracy and 90.6% sensitivity. General practitioners (GPs) benefitted from AI assistance, reaching 86.7% accuracy and 85.9% sensitivity, further improving diagnostic outcomes.

**Conclusions:**

This study highlights that AI models can effectively detect periodontal bone loss from panoramic radiographs, outperforming current diagnostic methods. The integration of AI into periodontal care offers faster, more accurate, and comprehensive treatment, ultimately improving patient outcomes and alleviating healthcare burdens.

## 1 Introduction

Periodontal diseases affect over a billion people worldwide and pose a significant public health challenge due to their high prevalence and potential to cause serious oral health issues if untreated (1). These conditions are primarily caused by bacterial infections, particularly from gram-negative anaerobes such as *Porphyromonas gingivalis*, *Treponema denticola*, and *Tannerella forsythia*. These bacteria trigger chronic inflammation and damage to the structures supporting the teeth, potentially leading to tooth loss. Additionally, periodontal diseases have been associated with systemic health conditions, including cardiovascular disease, diabetes, and respiratory disorders (2). Accurate and early diagnosis is essential for effective treatment; however, current diagnostic approaches, which rely heavily on clinical examinations and radiographic interpretation, have significant limitations (3). These methods are often time-consuming, subjective, and prone to variability due to factors such as practitioner experience and the complexity of periodontal structures (4, 5). As a result, diagnostic errors and inconsistencies are common, highlighting the need for more objective and efficient diagnostic tools.

Artificial intelligence (AI) has recently brought transformative changes to medicine, including the field of dentistry (6). Machine learning and deep learning algorithms, particularly convolutional neural networks (CNNs), have shown considerable promise in automating and improving diagnostic accuracy in medical imaging (7). In dental radiography, AI can provide rapid, accurate, and automated assessments of periodontal health, potentially overcoming the limitations of current methods (8). The integration of AI in dental diagnostics could revolutionize clinical practice by enabling early and precise diagnoses, improving patient outcomes, and streamlining treatment interventions (9). This shift toward AI-enhanced diagnostics aligns with broader trends in digital health and personalized medicine, offering a more efficient, cost-effective, and patient-centered approach to dental care. In addition, recent studies have successfully employed these techniques for periodontal diagnosis. For instance, CNNs, which excel in image processing, have been widely used for detecting periodontal bone loss from panoramic radiographs, showing high accuracy and sensitivity in classifying different stages of periodontitis (10). Support vector machines (SVM) and Decision trees (DT) models have also been explored for classifying periodontal disease, with SVM demonstrating solid performance in distinguishing between healthy and diseased tissues based on radiographic features (11). Furthermore, hybrid approaches combining CNNs with SVM or DT have shown promise in improving the precision of periodontal disease detection, as demonstrated by several studies in the field (12, 13). While this study utilizes YOLOv8, a state-of-the-art object detection model, it is essential to compare its performance with these traditional and hybrid AI techniques to assess its relative strengths and weaknesses. YOLOv8's ability to process images in real-time offers advantages in speed, but its performance in early-stage detection still requires improvement when compared to the more established CNN-based models, as noted in prior research (14). Additionally, methods such as Faster-RNN, which focus on sequence learning and pattern recognition, could potentially enhance the detection of periodontal disease progression in longitudinal studies (15). Thus, comparing YOLOv8 with these techniques would provide valuable insights into its applicability and potential for integration into clinical practice.

In recent years, the use of AI in periodontal disease detection has gained significant attention, particularly in analyzing panoramic radiographs. Kong et al. (10) employed a deep learning model with CNNs to detect periodontal disease, demonstrating the growing role of AI in this field. However, our study advances this by utilizing YOLOv8, a more sophisticated object detection model, which offers real-time processing and enhanced localization capabilities. Unlike Kong's model, YOLOv8 not only detects bone loss but also accurately segments critical anatomical landmarks, such as the cemento-enamel junction (CEJ) and alveolar bone levels, enabling more precise classification and staging of periodontitis (10). In comparison, Zhang et al. (11) used SVM to classify periodontal disease from radiographs, which showed good performance but was limited in processing the full complexity of radiographic images. Our YOLOv8 model, in contrast, offers more sophisticated real-time detection, including the quantification of bone loss per tooth—a feature absent in Zhang's approach (11). Similarly, Lee et al. (9) focused on hybrid AI models, combining CNNs with other machine learning algorithms, to detect periodontal disease. Their approach showed promise but relied heavily on manual feature extraction, which can be prone to error. Our use of YOLOv8 streamlines this process by eliminating manual extraction and providing end-to-end detection, increasing both speed and accuracy. Additionally, our model's ability to quantify bone loss and stage periodontitis sets it apart, providing a more robust diagnostic solution (9). Furthermore, the work of Li et al. (16, 17) also contributed to AI-driven periodontitis diagnosis, emphasizing interpretable models (16, 17). However, our study builds on their foundation by integrating real-time detection and classification, enabling more detailed analysis and improving clinical decision-making. This combination of advanced segmentation, real-time processing, and interpretability positions YOLOv8 as a powerful tool for enhancing periodontal disease diagnosis in clinical practice.

This study employs state-of-the-art AI technologies to address the critical challenges inherent in current periodontal diagnostics. Utilizing a dataset of 2,000 panoramic radiographs, we developed advanced CNNs and implemented the YOLOv8 model to precisely identify periodontal bone loss and assess the stages of periodontitis. The study aims to develop and validate AI-driven models to enhance diagnostic accuracy and efficiency, providing a more objective and consistent approach to periodontal disease detection.

## 2 Materials and methods

### 2.1 Study design

This retrospective study analyzed 2,000 panoramic radiographs from the Dental Department at Fang Hospital in Chiang Mai, Thailand. No intraoral examinations were performed as part of this study. The radiographs were collected between January 2015 and December 2023, using diagnostic codes from the HOSxP program (Bangkok Medical Software, Bangkok, Thailand). All images were captured using a consistent device and stored in the SIDEXIS Next Generation Program (Sirona, Bensheim, Germany). To maintain the quality and accuracy of the dataset, radiographs were excluded if they showed improper patient positioning, poor quality due to movement, rare bone morphologies, or if the alveolar bone loss in the affected area could not be accurately assessed. The study was approved by the Ethical Review Board of Fang Hospital (COA No. 03/2566) and the Ethics Committee for Research Involving Human Subjects at Mahasarakham University (No. 533-589/2023). Additionally, permission for data collection was granted by the director of Fang Hospital in Chiang Mai, Thailand (No. 0033.306/3674).

### 2.2 Inclusion and exclusion criteria

**Inclusion Criteria:**

1. **Age:** Participants aged 18 years and older to ensure that only fully erupted molars are included, while excluding erupting or unerupted molar teeth.
2. **Diagnosis:** Individuals diagnosed with periodontitis, as identified through diagnosis codes from the HOSxP Program (Bangkok Medical Software, Bangkok, Thailand).
3. **Radiograph Quality:** High-quality panoramic radiographs obtained from the SIDEXIS Next Generation Program (Sirona, Bensheim, Germany) and captured using a consistent device.
4. **Periodontal Classification** (18): The severity score reflects the attachment loss attributed solely to periodontitis, based on the most affected tooth.

**Severity of periodontitis** (Table 1):

1.1 Stage I: Interdental CAL of 1–2 mm and <15% radiographic bone loss.
1.2 Stage II: Interdental CAL of 3–4 mm and 15%–33% radiographic bone loss.
1.3 Stage III: Interdental CAL of ≥5 mm, bone loss to the middle third of root and beyond, and ≤4 teeth lost due to periodontitis.
1.4 Stage IV: Interdental CAL of ≥5 mm, bone loss to the middle third of root and beyond, and ≥5 teeth lost due to periodontitis.

**Table 1:** Staging of periodontal disease according to the 2018 classification criteria (18).

Periodontitis stage		Stage I	Stage II	Stage III	Stage IV
Severity	Interdental CAL at site of greatest loss	1 to 2 mm	3 to 4 mm	≥5 mm	≥5 mm
	Radiographic bone loss	Coronal third (<15%)	Coronal third (15% to 33%)	Extending to middle or apical third of the root	Extending to middle or apical third of the root
	Tooth loss	No tooth loss due to periodontitis		Tooth loss due to periodontitis of ≤4 teeth	Tooth toss due to periodontitis of ≥5 teeth
Complexity	Local	Maximum probing depth ≤4 mm Mostly horizontal bone loss	Maximum probing depth ≤5 mm Mostly horizontal bone loss	In addition to stage II complexity: Probing depth ≥6 mm Vertical bone loss ≥3 mm Furcation involvement Class II or III Moderate ridge defect	In addition to sage III complexity: Need for complex rehabilitation due to: Masticatory dysfunction Secondary occlusal trauma (tooth mobility degree ≥2) Severe ridge defect Bite collapse, drifting. flaring Less than 20 remaining teeth (10 opposing pairs)
Extent and distribution	Add to stage as descriptor	For each stage, describe extent as localized (<30% of teeth involved), generalized, or molar/incisor pattern			

**Exclusion Criteria:**

1. **Missing Radiographs:** Absence of panoramic radiographs in the SIDEXIS Next Generation Program.
2. **Image Quality:** Radiographs were excluded if they exhibited improper patient positioning, poor quality due to movement, uncommon bone morphologies, or if the alveolar bone loss in the affected area could not be accurately assessed**.**
3. **Panoramic radiographs of patients with craniofacial anomalies**, as these conditions may affect bone morphology.

### 2.3 Data collection

The dataset consisted of 2,000 panoramic radiographs from patients diagnosed with periodontitis, identified through diagnosis codes from the HOSxP Program (Bangkok Medical Software, Bangkok, Thailand). Radiographs were excluded if they exhibited improper positioning, suboptimal image quality, or rare bone morphologies.

### 2.4 Data handling and ethical considerations

Data were anonymized to protect patient confidentiality. Ethical guidelines were strictly followed throughout the study to ensure compliance with institutional and regulatory standards.

### 2.5 Image enhancement

**Figure 1:**
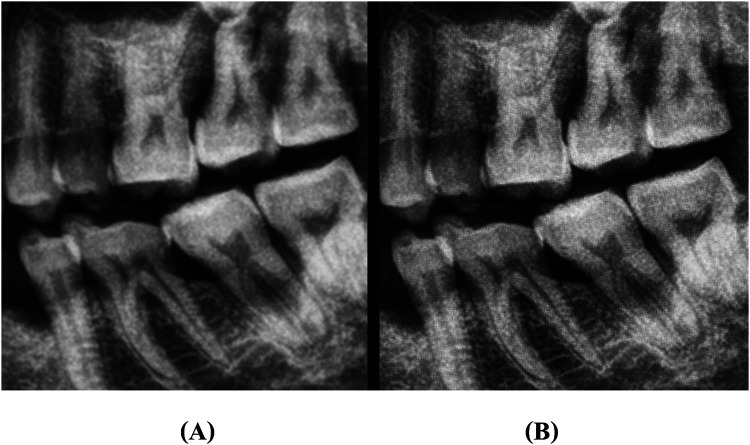
An illustration of image sharpening: **(A)** represents original image, and **(B)** represents sharpened image.

**Figure 2:**
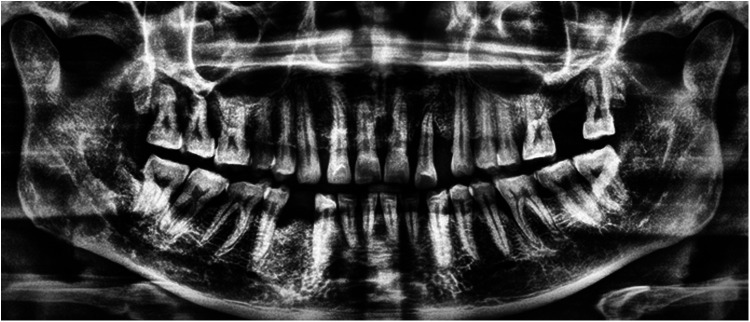
An illustrative of image contrast adjustment using histogram equilibrium.

**Figure 3:**
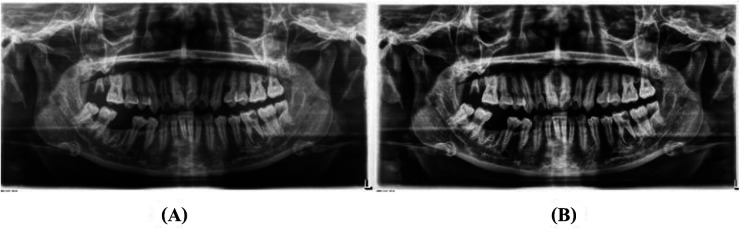
An illustration of Gaussian filtering: **(A)** represents original image, and **(B)** represents the image after preprocessing.

Image preprocessing involved several enhancement techniques to improve the clarity and quality of the radiographs. This included (19):

1. **Image Sharpening**: Enhancing edges to make pixel boundaries more distinct and improve visual interpretability (Figure 1).
2. **Contrast Adjustment**: Using histogram equalization to balance brightness levels and distinguish target areas from the background (Figure 2).
3. **Gaussian Filtering**: Applying a 3 × 3 kernel matrix to reduce noise and smooth the images (Figure 3).

### 2.6 Data labeling

**Figure 4:**
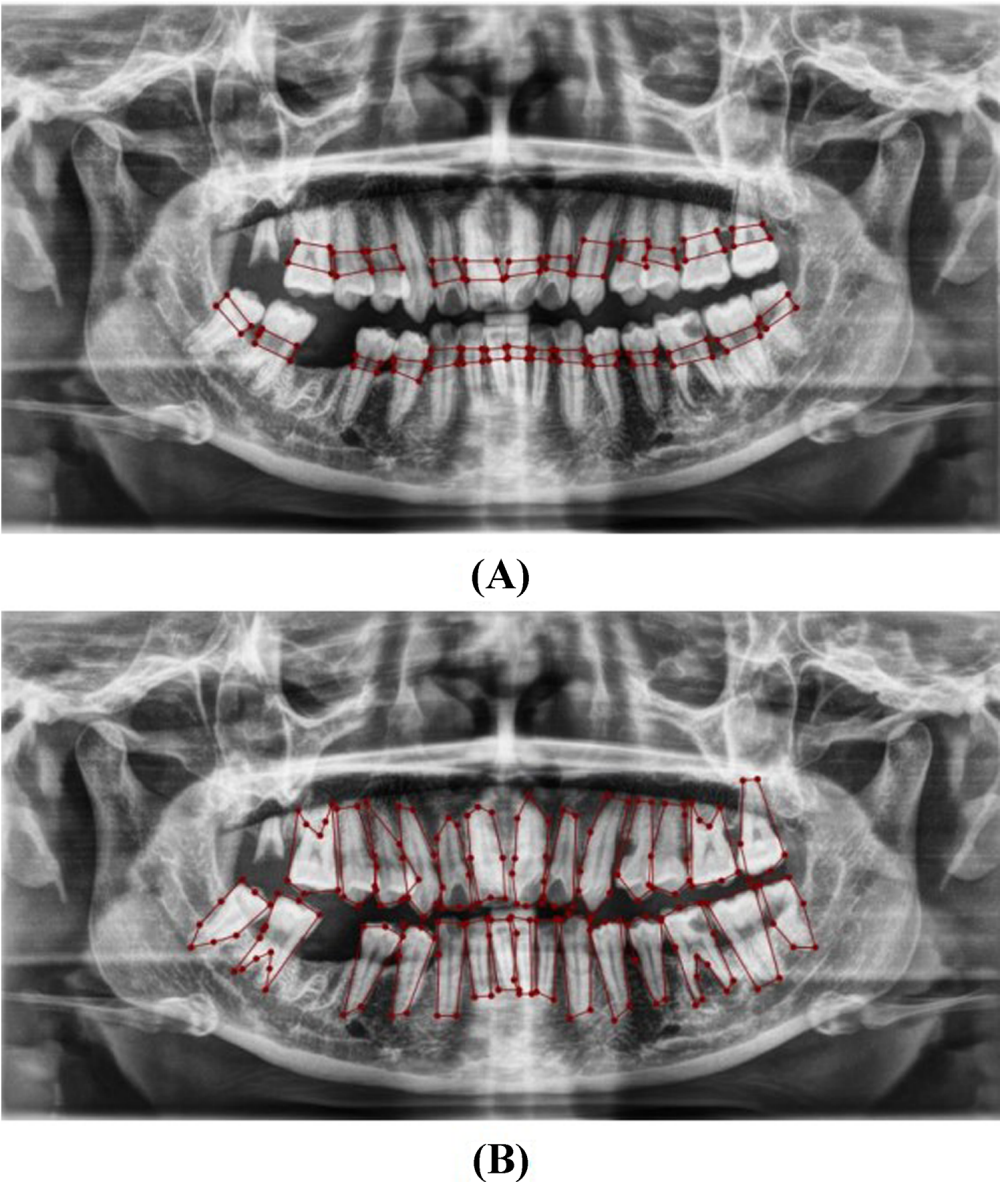
Image showing the distance between the CEJ and the alveolar bone crest **(A)** and teeth **(B)**, labeled using LabelMe.

Labeling of the images was performed using the LabelMe tool for object segmentation and Labelme2yolo for data conversion. Annotations focused on the cemento-enamel junction (CEJ) and the alveolar bone crest (20) (Figure 4). Recognizing the critical role of labeled data in supervised machine learning, this research utilized LabelMe for object segmentation and Labelme2yolo to convert the data into a format ready for training.

### 2.7 AI model development

**Figure 5:**
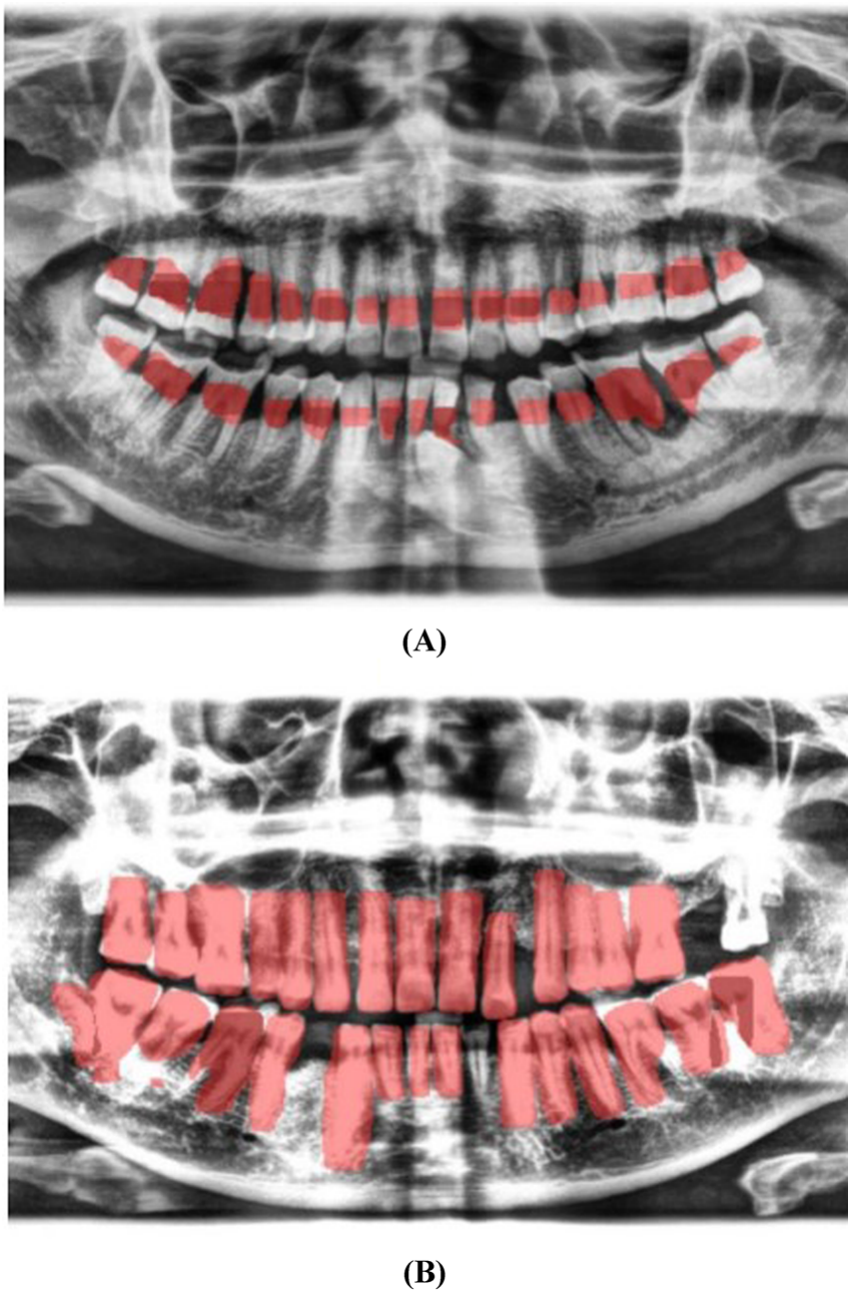
Image showing the predicted area between the CEJ and the alveolar bone crest **(A)**, and teeth segmentation **(B)**.

**Figure 6:**
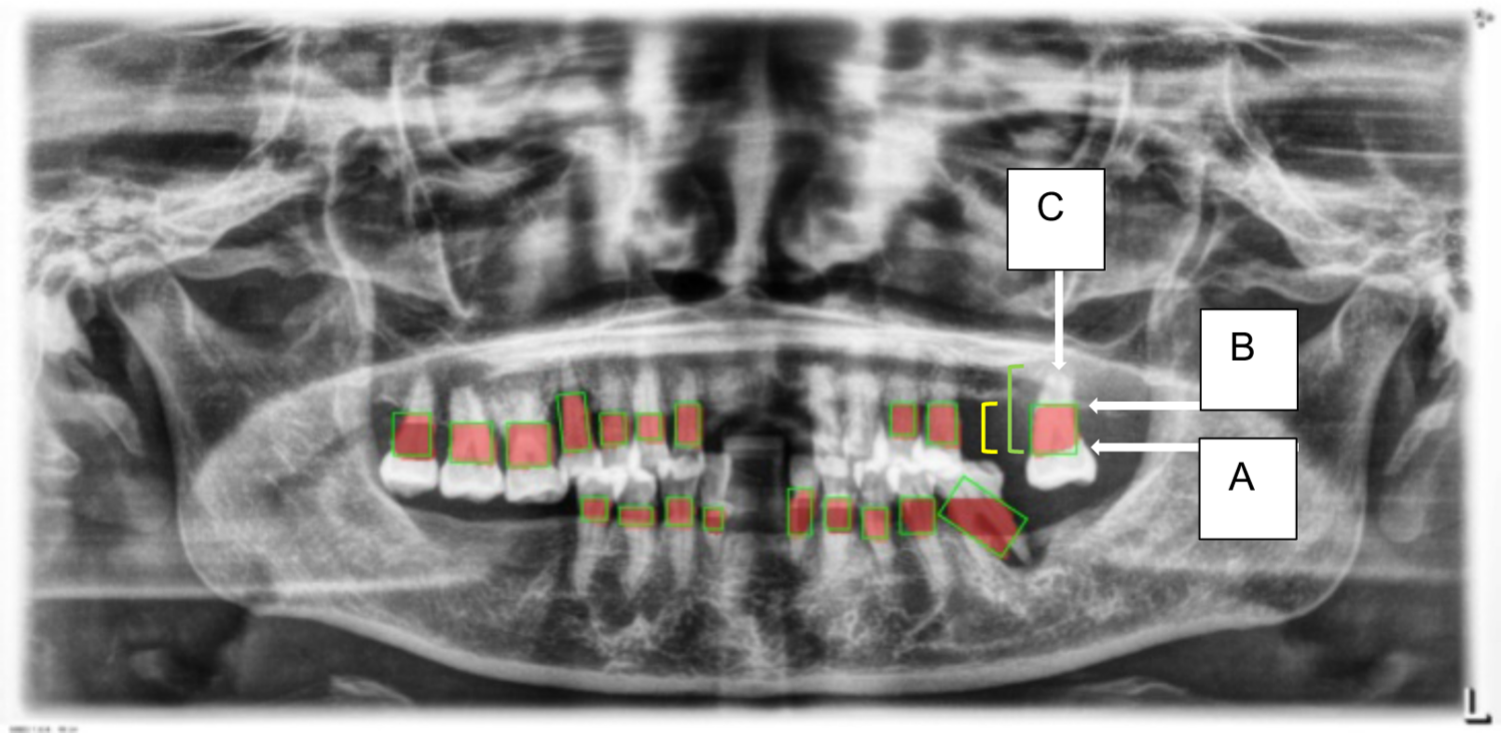
This figure illustrates an example of radiographic bone loss. The color coding for the lines is as follows: yellow denotes the distance from the cemento-enamel junction (CEJ) **(A)** to the alveolar bone crest level **(B)**, green represents the distance from the CEJ to the root apex **(C)**, and red boxes highlight the distance from the CEJ to the alveolar bone crest level.

**Figure 7:**
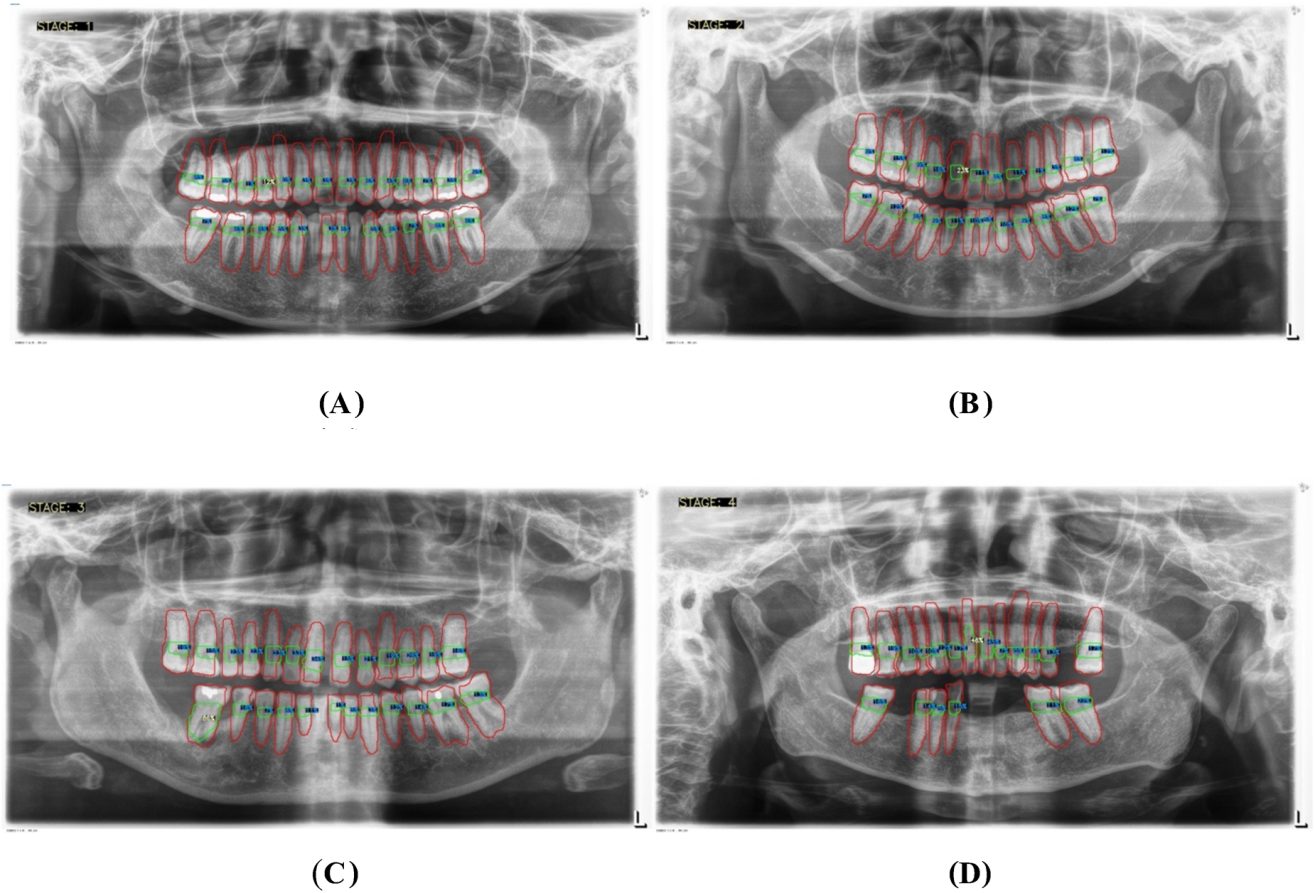
Panoramic x-ray images illustrating the threshold percentages for periodontitis severity: **(A)** represents stage I, **(B)** represents stage II, **(C)** represents stage III, and **(D)** represents stage IV.

**Figure 8:**
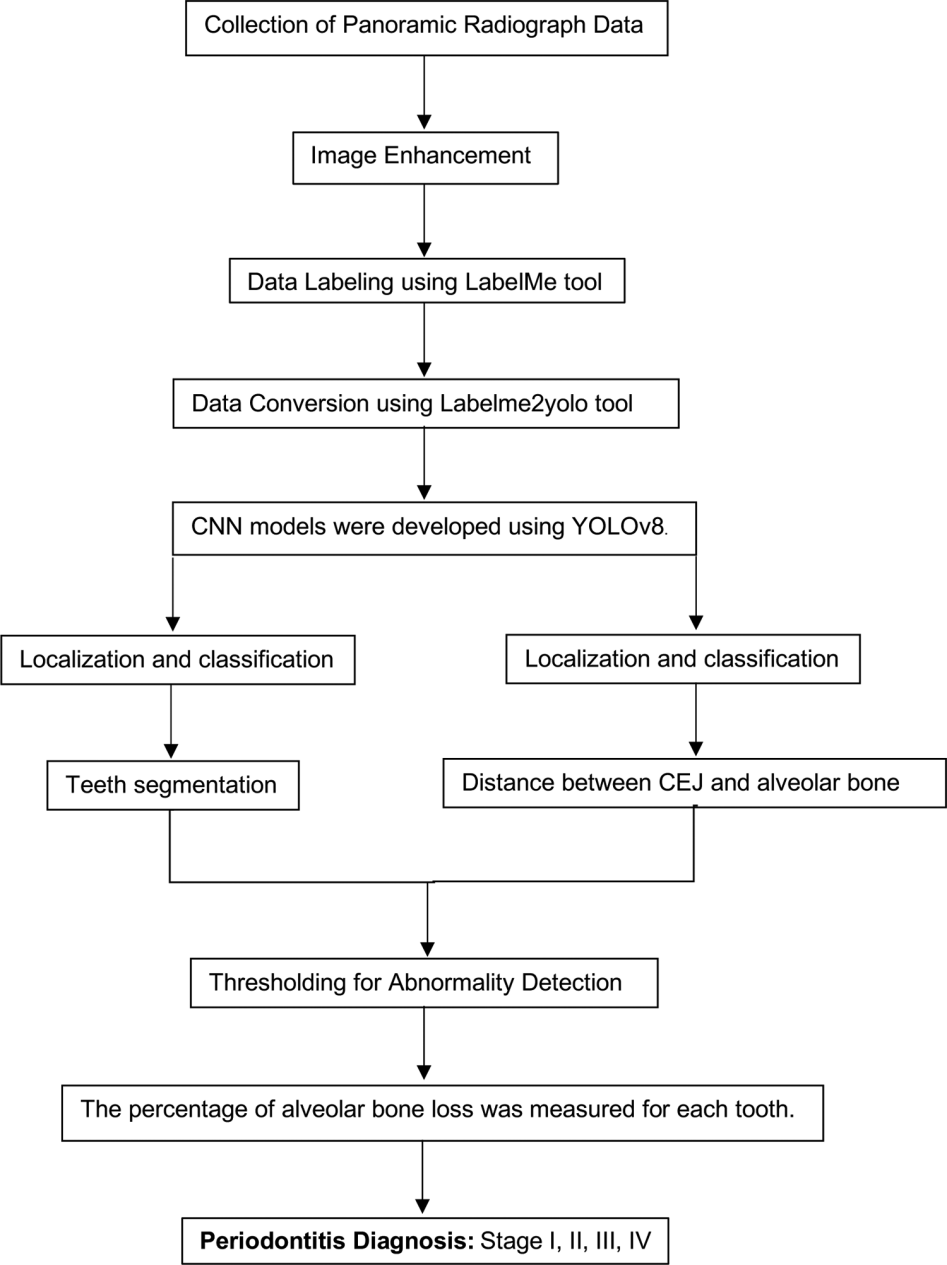
Comprehensive workflow for a hybrid framework utilizing deep learning architecture to detect and classify the stages of periodontitis.

A CNN model was developed utilizing YOLOv8 (You Only Look Once version 8) for identifying regions of interest within the radiographs (21, 22). The development process followed a structured workflow:

1. **Data Segmentation**: The dataset was divided into training, validation, and test sets in a 70:10:20 ratio to ensure balanced model evaluation.
2. **Training Setup**: The model was trained on an Intel Core i7–8,700 K CPU with 16 GB RAM, a Nvidia GeForce RTX2080 GPU equipped with 8 GB of video memory, and implemented using CUDA Toolkit 9.0, CUDNN V11.7, and Python 3.11.5. The primary hyperparameters used were the learning rate (set at 0.001), batch size (64), and the number of epochs (100). The evaluation metrics included accuracy, sensitivity, specificity, precision, and F1-score, with mAP50 (mean average precision at a 50% intersection-over-union threshold) also being calculated to assess model performance.
3. **Localization and Classification**: The model was trained to identify and localize the region between the cemento-enamel junction (CEJ) and the alveolar bone crest, producing bounding boxes or heat maps for further analysis (Figure 5).
4. **Thresholding for Abnormality Detection (Severity of Periodontitis):** A thresholding mechanism will be devised to determine the extent of abnormality based on the width of the gap between the CEJ and the bone structure (Figure 6). Teeth with gaps exceeding the predefined threshold (e.g., >2 mm) will be flagged as abnormally positioned. To calculate the percentage of bone loss, use the formula (18):
  $$\mathrm{Percentage}\;\mathrm{of}\;\mathrm{bone}\;\mathrm{loss}=\frac{(\mathrm{CEJ}-\mathrm{Alveolar}\;\mathrm{bone}\;\mathrm{crest})-2\;\mathrm{mm}}{(\mathrm{CEJ}-\mathrm{Root}\;\mathrm{apex})-2\;\mathrm{mm}}\times 100$$
  For periodontal diagnosis, the assessments involved calculating the percentage of alveolar bone loss for each tooth in the radiographs. The stage of periodontitis for each patient was determined based on the greatest bone loss observed across all teeth, which was then used to assign the appropriate periodontitis stage as follows (Figure 7):
  • **Stage I**: Bone loss of less than 15% visible in x-rays.
  • **Stage II**: Bone loss ranging between 15% and 33% visible in x-rays.
  • **Stage III**: Bone loss extending beyond the middle third of the root, with up to 20 teeth remaining.
  • **Stage IV**: Similar to Stage III, with bone loss extending beyond the middle third of the root, but with fewer than 20 teeth remaining.
5. Comprehensive Workflow (Figure 8)

### 2.8 Model evaluation and validation

A confusion matrix was employed to evaluate the model's predictive accuracy, detailing True Positives (TP), True Negatives (TN), False Positives (FP), and False Negatives (FN). Key performance metrics such as Sensitivity (Recall), Specificity, F1-Score, Accuracy, and Precision were calculated to assess the model's overall effectiveness.

### 2.9 Clinical implementation

Based on the research conducted by Lee et al. (9), the agreement values between the convolutional neural network (CNN) and periodontists were reported. For premolars, the agreement values were 0.828 and 0.797, respectively, and for molars, the values were 0.734 and 0.766, respectively. When calculating the sample size, the researcher set *d*, the deviation of the agreement values from previous studies at 25%. The results are shown in Table 2:

**Table 2:** Sample size calculation for molar cases with adjusted error rates (25%).

Formula	P (CNN)	P (periodontists)	k_o_	Deviation (%)	k_1_	Sample Size (n)
1.	0.734	0.766	0.766	25	0.9575	83

The sample size calculation is as follows (23, 24);

$$\begin{array}{cc} n & {=\left[\frac{z_{1}-\frac{\alpha }{2}\sqrt{Q_{0}}+z_{1}-\beta \sqrt{Q_{1}}}{k_{1}-k_{0}}\right]}^{2}\\ Q & ={\left(1-\pi_{e}\right)}^{-4}\{\sum_{i}\pi_{ii}{\left[(1-\pi_{e})-(\pi_{\cdot i}+\pi_{i\cdot })(1-\pi_{0})\right]}^{2}\\ & \;+\;(1-\pi_{0}){]}^{2}\sum \sum_{i\neq j}\pi_{ij}{\left(\pi_{\cdot i}+\pi_{\;j\cdot }\right)}^{2}-{\left(\pi_{0}\pi_{e}-2\pi_{e}+\pi_{0}\right)}^{2}\} \end{array}$$

When *n* is the sample size for estimating agreement:

$k_{1}$ is the alternative hypothesis value of the Kappa statistic.

$k_{0}$ is the null hypothesis value of the Kappa statistic.

$\pi_{e}$ is the probability that Rater 1 gives a positive result.

$\pi_{0}$ is the probability that Rater 2 gives a positive result.

In the clinical phase of this study, we initially calculated a sample size of 83 panoramic x-ray images, considering a 25% adjusted error rate. Ultimately, 90 images were used to assess diagnostic agreement between the AI, periodontist, general practitioner (GP), and an expert. Moreover, the panoramic radiographs used in this study were not accompanied by definitive clinical diagnoses. Instead, they served as an initial diagnostic tool for periodontitis, based on screening conducted during clinical exams and the analysis of the panoramic radiographs. These findings were subsequently recorded in the periodontal diagnosis code within the HOSxP program at the hospital. The expert periodontist, with over 10 years of experience in Periodontology, was regarded as the gold standard. One periodontist, one GP, and one periodontitis expert evaluated the same set of radiographs to compare their assessments, enabling us to measure diagnostic agreement using the weighted Cohen's Kappa statistic. Statistical analysis was conducted using Stata 17.0 software (StataCorp, College Station, TX, USA), with a *p*-value of 0.05 considered statistically significant.

## 3 Results

The demographic characteristics of the patients, as outlined in Table 3, provide a foundation for understanding the study's cohort. Table 4 then showcases the performance of the AI models, with the teeth segmentation model achieving a notable 0.97 accuracy, 0.90 sensitivity, and 0.96 specificity, effectively distinguishing between true positives and negatives. Its precision and F1-score, both at 0.80, reflect a balanced capacity for accurate predictions and recall. In comparison, the CEJ and alveolar bone crest level segmentation model demonstrated superior performance, with 0.98 accuracy, 1.0 sensitivity, 0.90 precision, 0.90 F1-score, and 0.98 specificity. The model also achieved a mAP50 of 0.995, underscoring its enhanced precision and overall effectiveness. These findings highlight the high efficacy of both AI models, particularly in detecting periodontal bone loss, with the CEJ and alveolar bone crest level model excelling in sensitivity and accuracy, positioning it as a highly effective tool for precise periodontal diagnosis. This underscores the transformative potential of AI models in automating and enhancing the accuracy of periodontal disease detection.

**Table 3:** The demographic data of the patients.

Sex	Numbers of patients	Mean age (years)
Male	823	47.04
Female	1,177	45.27

**Table 4:** The AI models developed achieved the following scores (25).

	Teeth segmentation model	CEJ and alveolar bone crest level segmentation model
Precision	0.80 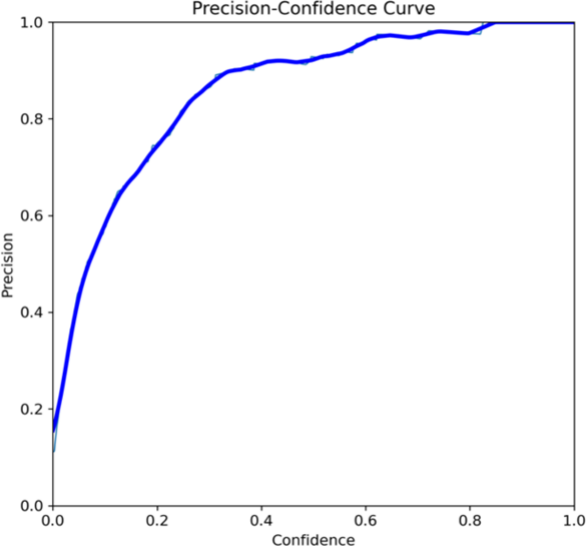	0.90 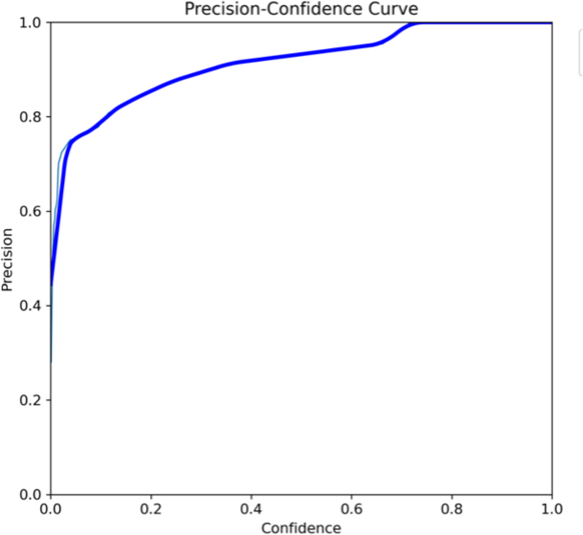
F1	0.80 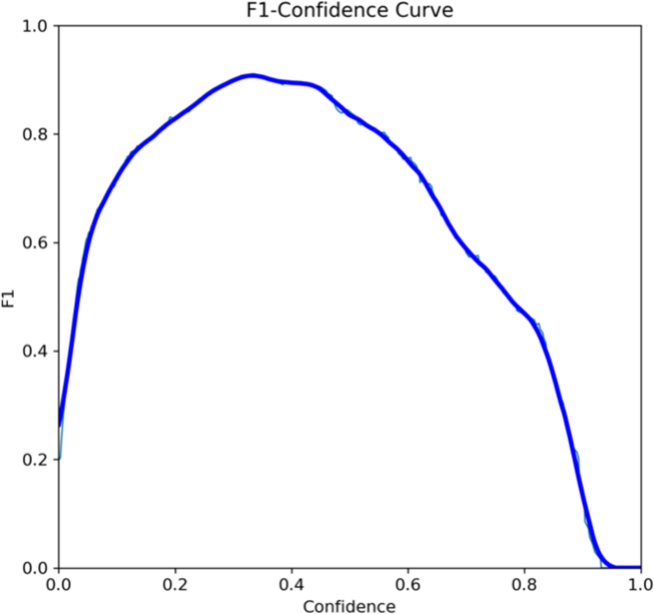	0.90 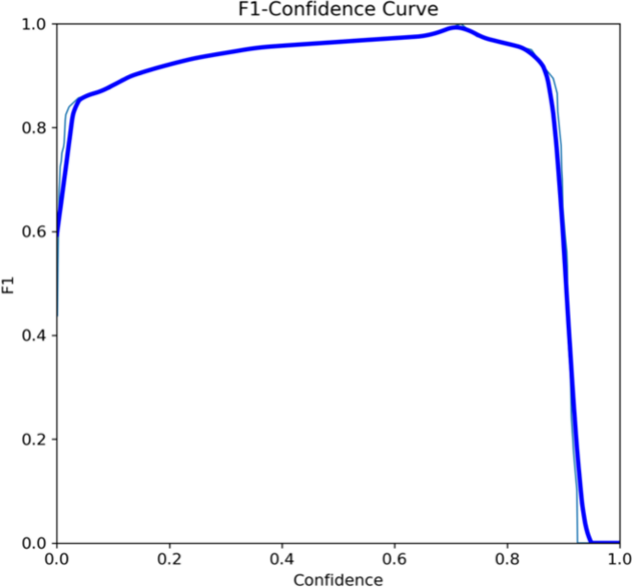
Sensitivity	0.90 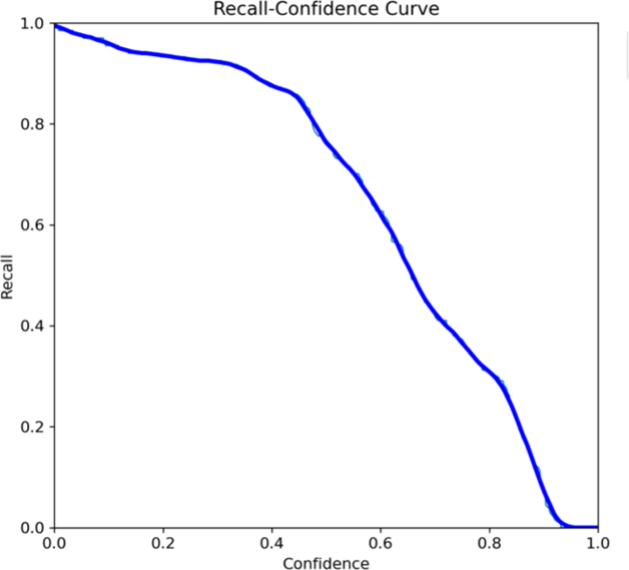	1.0 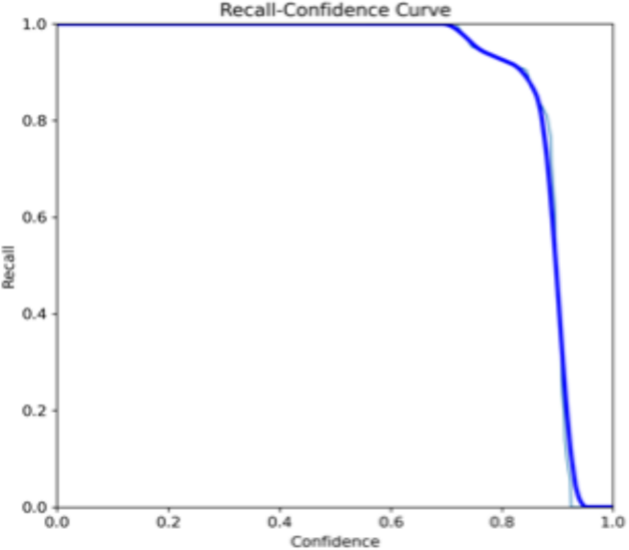
Specificity	0.96 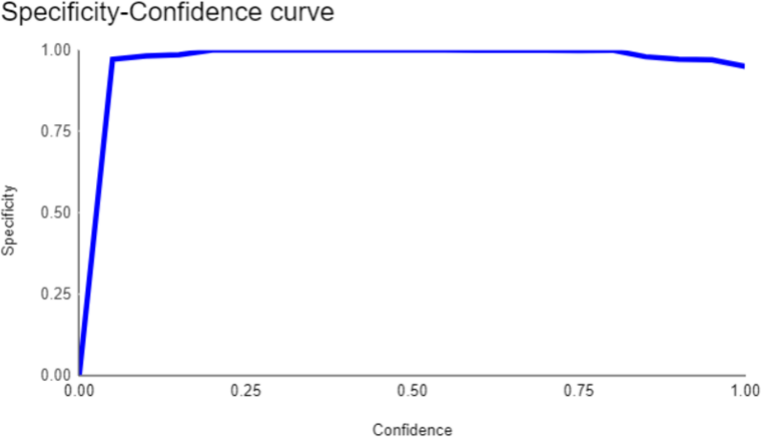	0.98 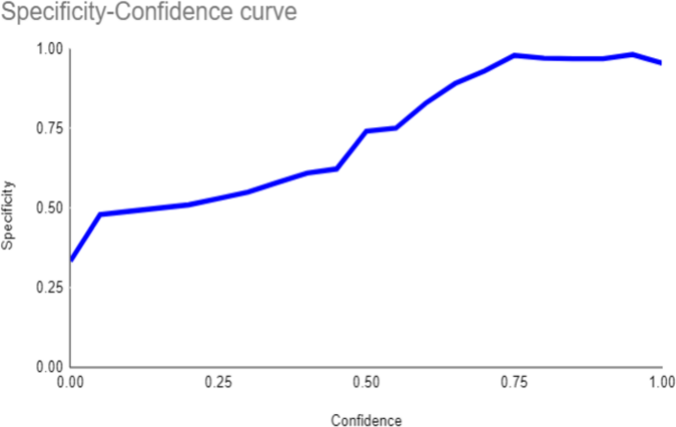
Accuracy	0.97 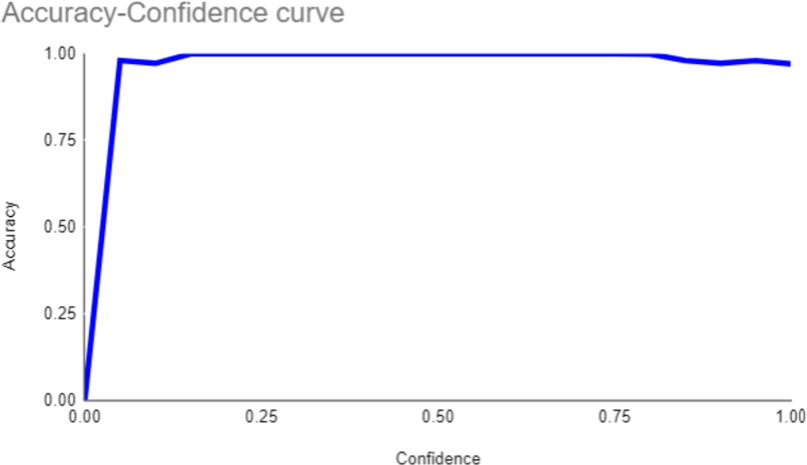	0.98 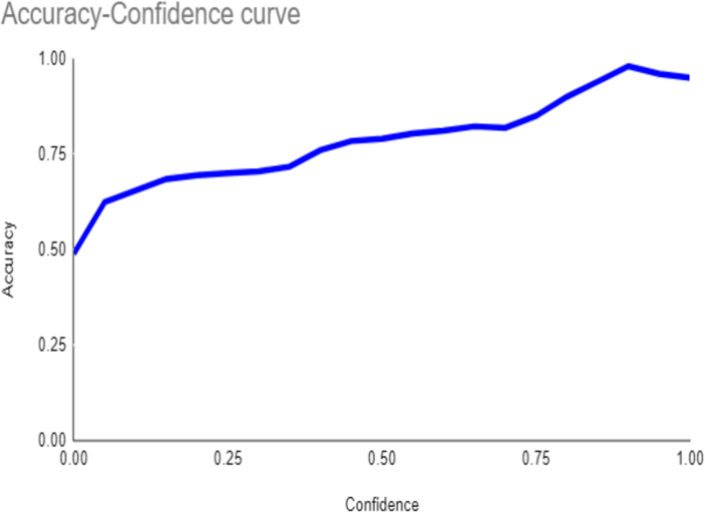
mAP50	0.92	0.995
Confusion matrix	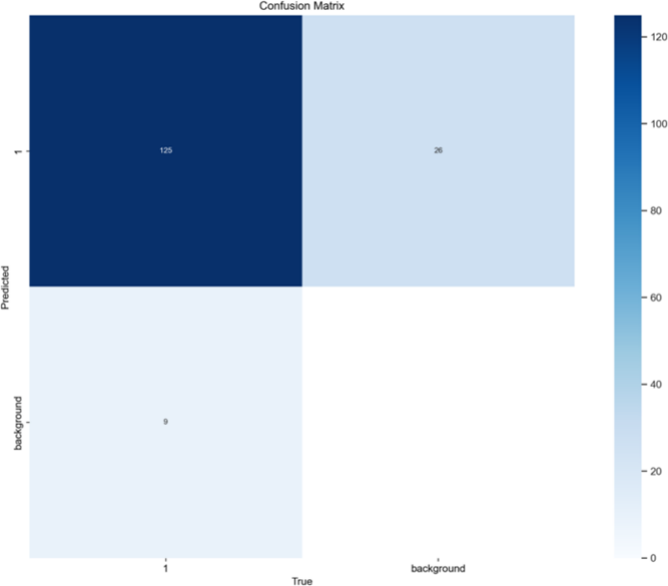	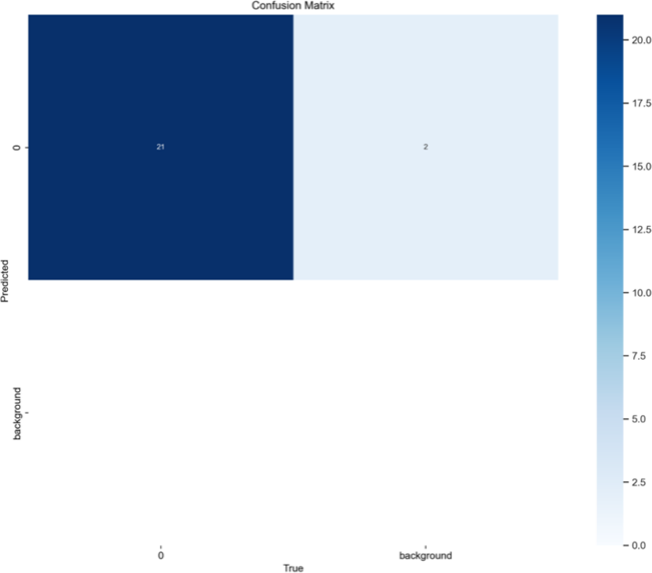

**Figure 9:**
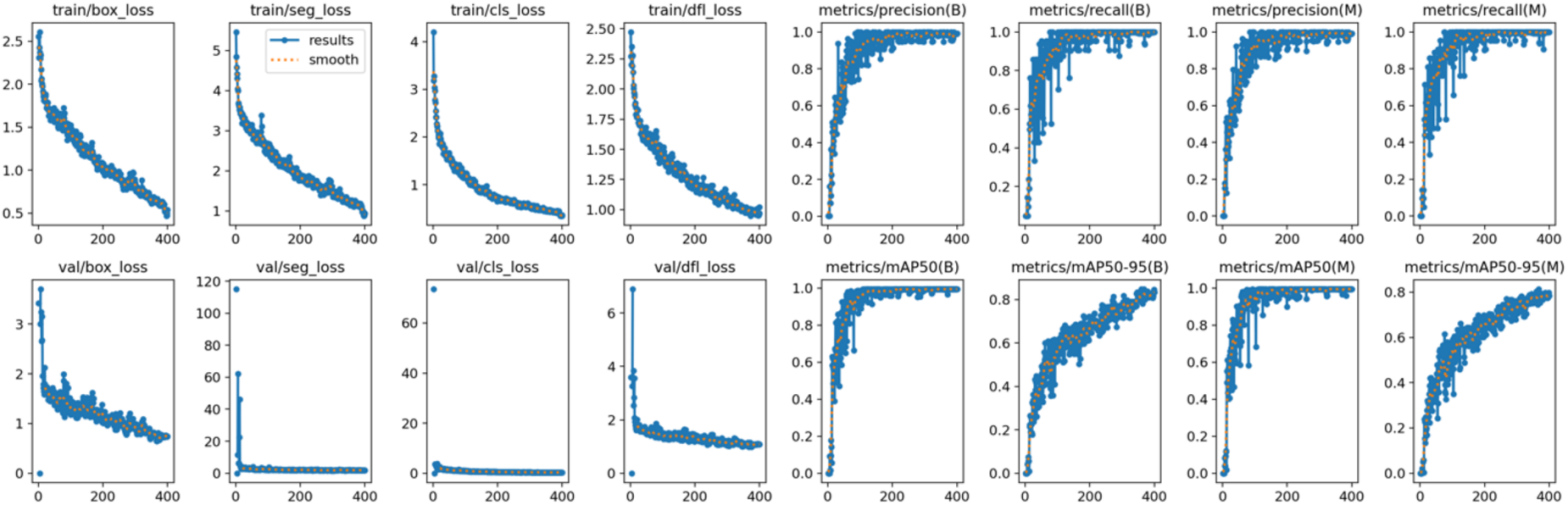
Results from the CEJ and bone level segmentation model. This figure displays the performance of the model in accurately identifying and segmenting the cemento-enamel junction (CEJ) and alveolar bone levels from panoramic radiographs, demonstrating its ability to delineate critical anatomical landmarks for the assessment of periodontal disease stages. The segmentation results provide valuable data for evaluating the extent of bone loss, a key indicator in diagnosing periodontitis.

**Figure 10:**
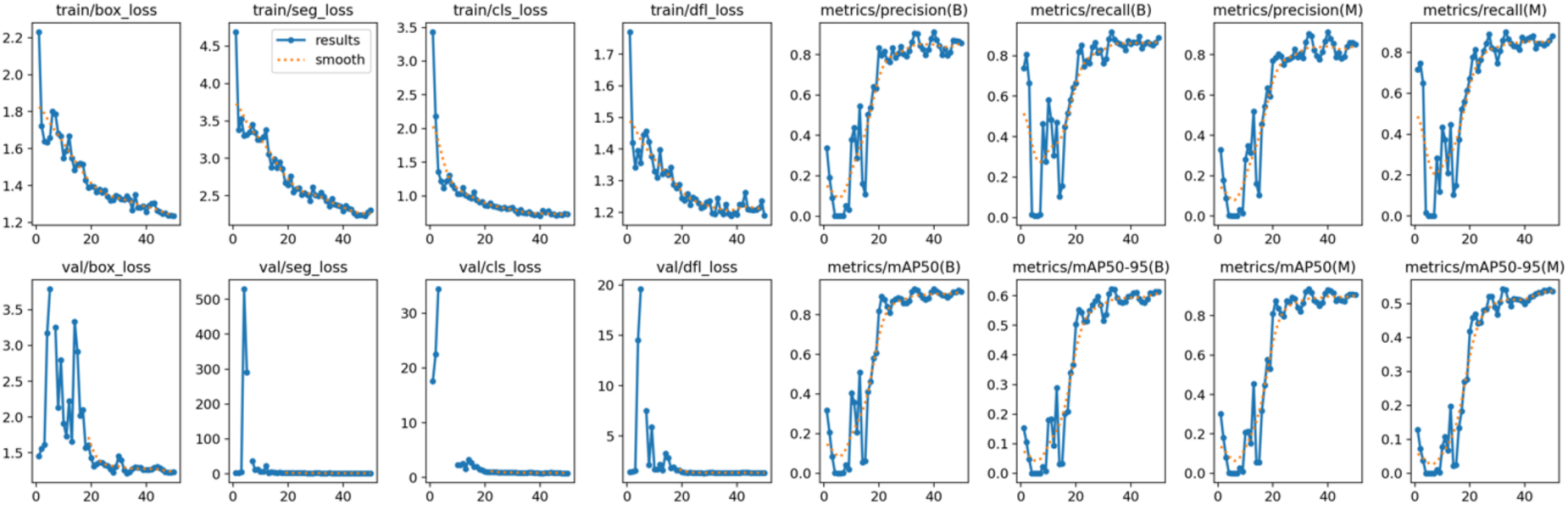
The results of the teeth segmentation model, showing the performance of the AI model across various metrics, including loss and precision-recall. The figure demonstrates the accuracy of the model in segmenting individual teeth from the panoramic radiographs during training, validation, and evaluation, with the corresponding metrics presented for each phase of the process.

Both the CEJ and bone level segmentation model (Table 5) and the teeth segmentation model (Table 6) demonstrated strong performance in accurately classifying relevant areas in panoramic radiographs. In Table 5, the CEJ and bone level model correctly predicted 18,385 instances, with only 234 false positives, indicating high precision. The model also exhibited strong recall, with minimal false negatives (11). Similarly, the teeth segmentation model (Table 6) performed well, accurately identifying 983 teeth instances and 18,687 true negatives. However, it had a slightly higher false positive rate (589), where non-teeth areas were incorrectly classified as teeth. Despite the higher false positive rate in the teeth model, both models exhibited high accuracy and efficiency in their respective tasks, with low false negative rates and a strong ability to differentiate between positive and negative classes in their predictions. Moreover, the training results from the CEJ and bone level segmentation model and the teeth segmentation model are presented in Figures 9, 10, respectively.

**Table 5:** Confusion matrix for the CEJ and bone level segmentation model.

	Actual value		
Predicted value		Positive	Negative
	Positive	TP:508	FP:234
	Negative	FN:11	TN:17877

True Positive (TP): Correctly identified areas indicating bone loss.

True Negative (TN): Correctly identified areas without bone loss.

False Positive (FP): Areas incorrectly labeled as having bone loss when none is present.

False Negative (FN): Areas with bone loss that were incorrectly identified as normal.

**Table 6:** Confusion matrix for the teeth segmentation model.

	Actual value		
Predicted value		Positive	Negative
	Positive	TP:983	FP:589
	Negative	FN:11	TN:18687

True Positive (TP): Correctly identified areas indicating bone loss.

True Negative (TN): Correctly identified areas without bone loss.

False Positive (FP): Areas incorrectly labeled as having bone loss when none is present.

False Negative (FN): Areas with bone loss that were incorrectly identified as normal.

In the clinical implementation, we calculated a sample size of 83 panoramic x-ray images with an adjusted error rate of 25%, but we used 90 images to compare the accuracy of the AI model, general practitioner (GP), and periodontist against expert periodontist. The demographic data of the patients are presented in Table 7. Table 8 reveals the AI model achieved the highest accuracy (94.4%) and perfect sensitivity (100%), indicating its ability to detect all positive cases but struggled with specificity (0%), meaning it had difficulty ruling out false positives. Periodontist demonstrated strong overall performance with 91.1% accuracy, 90.6% sensitivity, and perfect specificity (100%), while GP showed slightly lower accuracy (86.7%) and sensitivity (85.9%) but also achieved perfect specificity (100%). The AI model's high sensitivity makes it effective at identifying true positives, though it requires human oversight for confirming negatives, as both periodontist and GP performed more consistently in terms of both sensitivity and specificity. The distribution of results among expert periodontist, periodontist, GP, and the AI model is illustrated in Table 9.

**Table 7:** Demographic data of patients used to compare the accuracy percentage of the AI model, general practitioners (GP), and periodontists with expert periodontist.

Sex	Numbers of patients	Mean age (years)
Male	42	44.29
Female	48	43.56

**Table 8:** The diagnostic performances of the AI model, general practitioner (GP), and periodontist with expert periodontist.

Test	Accuracy %	Sensitivity %	Specificity%	PPV %	NPV %
	(95% CI)	(95% CI)	(95% CI)	(95% CI)	(95% CI)
Periodontist	91.1	90.6	100	100	38.5
	(83.2–96.1)	(82.–95.8)	(47.8–100)	(95.3–100)	(13.9–68.4)
GP	86.7	85.9	100	100	29.4
	(77.9–92.9)	(76.6–92.5)	(47.8–100)	(95.1–100)	(10.3–56)
AI	94.4	100	0	94.4	0
	(87.5–98.2)	N/A	N/A	N/A	N/A

PPV, positive predictive value; NPV, negative predictive value; CI, confident interval.

**Table 9:** The distribution of result by expert periodontist, periodontist, general practitioner (GP) and AI model.

	Expert									
	0		1		2		3		4	
	*n*	(%)	*n*	(%)	*n*	(%)	*n*	(%)	*n*	(%)
Periodontist										
0	5	(5.6)	1	(1.1)	6	(6.7)	1	(1.1)	0	(0.0)
1	0	(0.0)	4	(4.4)	8	(8.9)	0	(0.0)	0	(0.0)
2	0	(0.0)	0	(0.0)	20	(22.2)	8	(8.9)	0	(0.0)
3	0	(0.0)	0	(0.0)	2	(2.2)	34	(37.8)	0	(0.0)
4	0	(0.0)	0	(0.0)	0	(0.0)	0	(0.0)	1	(1.1)
GP										
0	5	(5.6)	4	(4.4)	8	(8.9)	0	(0.0)	0	(0.0)
1	0	(0.0)	1	(1.1)	14	(15.6)	3	(3.3)	0	(0.0)
2	0	(0.0)	0	(0.0)	11	(12.2)	15	(16.7)	0	(0.0)
3	0	(0.0)	0	(0.0)	3	(3.3)	22	(24.4)	0	(0.0)
4	0	(0.0)	0	(0.0)	0	(0.0)	3	(3.3)	1	(1.1)
AI										
1	0	(0.0)	1	(1.1)	0	(0.0)	0	(0.0)	0	(0.0)
2	4	(4.4)	0	(0.0)	29	(32.2)	6	(6.7)	0	(0.0)
3	1	(1.1)	4	(4.4)	6	(6.7)	35	(38.9)	1	(1.1)
4	0	(0.0)	0	(0.0)	1	(1.1)	2	(2.2)	0	(0.0)

0 represents non-periodontitis, 1 represents periodontitis stage I, 2 represents periodontitis stage II, 3 represents periodontitis stage III, and 4 represents periodontitis stage IV.

Furthermore, we evaluated the diagnostic agreement between three different raters—AI, periodontist, and general practitioner (GP)—and an expert, considered the gold standard. We used the weighted Cohen's kappa statistic to measure the level of agreement beyond chance. The statistical significance of these weighted kappa coefficients was tested at an alpha level of 0.05. The weighted Cohen's kappa coefficients are presented in Table 10.

**Table 10:** The weighted Cohen's kappa coefficient for the comparisons of the AI model, general practitioners (GP), and periodontist with expert periodontist.

	Agreement (%)	Kappa (95% CI)		*p*-value
Periodontist vs. Expert	90.1	0.634	(0.621–0.694)	<0.001
GP vs. Expert	83.1	0.429	(0.298–0.542)	<0.001
AI vs. Expert	90.0	0.445	(0.398–0.471)	<0.001

From Table 10, the study reveals that the evaluations made by periodontist showed a high level of agreement with those of the expert, evidenced by a Cohen's kappa coefficient of 0.634 (95% CI: 0.621–0.694, *p*-value <0.001). The assessments by general practitioner (GP) demonstrated a moderate level of agreement with the expert, with a Cohen's kappa coefficient of 0.429 (95% CI: 0.298–0.542, *p*-value <0.001). Similarly, the AI model's evaluations also exhibited a moderate level of agreement with the expert, reflected by a Cohen's kappa coefficient of 0.445 (95% CI: 0.398–0.471, *p*-value <0.001). These results underscore the AI model's potential in achieving diagnostic consistency comparable to that of human practitioners, albeit at a moderate agreement level.

## 4 Discussions

The periodontology field updated its classification system in 2018, focusing now on the percentage of alveolar bone loss to assess disease severity (26). Challenges in current periodontitis diagnosis include error risks due to a scarcity of experienced clinicians, limited analysis time for radiographs, and mandatory reporting, affecting care quality, cost, and efficiency (27). These issues have led to discussions on creating supportive diagnostic tools. This study highlights the challenges of existing diagnostic methods and explores future technological solutions and research directions to address these limitations. In recent years, AI has begun to flourish in dentistry, offering a range of applications from diagnostics and decision-making to treatment planning and predicting outcomes. Additionally, AI tools for dental applications are becoming increasingly sophisticated, precise, and dependable, with research extending across all dental disciplines (28).

The present study found that the teeth segmentation model achieved sensitivity, specificity, F1, precision, and accuracy scores of 0.9, 0.96, 0.8, 0.8, and 0.97, respectively. In contrast, the CEJ and alveolar bone crest level segmentation model attained scores of 1, 0.98, 0.9, 0.9, and 0.98, respectively.

These results indicate that both AI models are highly effective, particularly in detecting periodontal bone loss. The CEJ and alveolar bone crest level segmentation model outperformed the teeth segmentation model, especially in terms of sensitivity and accuracy, making it an excellent tool for precise periodontal diagnosis. This demonstrates the potential of AI models in automating and improving the accuracy of periodontal disease detection.

In recent years, artificial intelligence (AI) has emerged as a promising tool in the field of periodontal disease detection, offering significant advancements over current diagnostic methods (28, 29). In the present study, the YOLOv8 model was used to analyze panoramic radiographs and segment crucial areas such as the teeth, cemento-enamel junction (CEJ), and alveolar bone levels, demonstrating superior performance in detecting periodontal bone loss compared to human experts, including periodontists and general practitioners (GPs). The accuracy of the AI model (94.4%) and its perfect sensitivity (100%) highlight its potential as a powerful diagnostic aid. However, to ensure comprehensive assessment and further validation, a broader comparison with other AI techniques and methods is essential. Previous research has demonstrated the use of various machine learning techniques such as convolutional neural networks (CNNs), support vector machines (SVM), decision trees (DT), and hybrid approaches in periodontal disease detection. For instance, CNNs have shown great potential in image processing and detecting periodontal bone loss with high sensitivity and accuracy, often outperforming current methods in classifying periodontal stages (10, 11). Additionally, hybrid models combining CNNs with SVM or DT have shown improved precision and accuracy by leveraging the strengths of each technique (12, 13). Furthermore, methods such as Faster RNN, which focus on sequence learning, can potentially enhance the detection of periodontal disease progression by capturing temporal patterns in longitudinal datasets (15). While the YOLOv8 model used in this study demonstrated excellent real-time processing capabilities, it requires further refinement in early-stage detection compared to CNN-based models, which have shown greater sensitivity in identifying mild stages of periodontitis (14, 30). Overall, comparing YOLOv8 with these established AI techniques, such as CNN and hybrid models, could provide valuable insights into the strengths and weaknesses of each approach, ensuring a more robust and versatile tool for clinical practice in periodontal diagnostics.

Despite the recent surge in publications on dental AI, comparing these studies is challenging due to discrepancies in study design, data distribution (training, testing, and validation sets), and performance metrics (accuracy, sensitivity, specificity, F1 score, precision). Many articles do not fully report these critical details. However, accuracy emerged as the most commonly referenced indicator of model performance in the studies, with detection rates for periodontal bone loss ranging between 0.76 and 0.93 (31–34). This is consistent with the findings of this study, which reported accuracy scores of 0.97 for the teeth segmentation model and 0.98 for the CEJ and alveolar bone crest level segmentation model. These accuracy scores are higher than those reported in all previous studies. The dataset of dental panoramic x-ray images used in various studies ranged from 100 to 2,276 images (31–37), with only one study employing a significantly larger dataset of 12,179 images (38). In this study, a dataset of 2,000 dental panoramic x-ray images was used, yet the accuracy rate remained high. Furthermore, many studies have aimed to detect periodontal bone loss on dental panoramic x-ray images. Specifically, Chang et al. (39) advanced study, which sought to classify stages of periodontitis following the latest periodontal classification, found that the automatic method had a Pearson correlation coefficient of 0.73 with radiologist diagnoses for the entire jaw and an intraclass correlation of 0.91 for the entire jaw (39). Similarly, Jiang et al. (33) revealed creating a deep learning model to evaluate and categorize the stages of periodontitis, achieving an overall model accuracy of 0.77 (33). Additionally, our study uniquely detects periodontal bone loss, classifies the stage of periodontitis, and identifies the percentage of bone loss for each tooth, aiding in prognosis evaluation. This novel innovation has not been previously achieved.

We evaluated the diagnostic agreement between three different raters—AI, periodontist, and general practitioner (GP)—and an expert periodontist, considered the gold standard. We used the weighted Cohen's kappa statistic to measure the level of agreement beyond chance. The statistical significance of these weighted kappa coefficients was tested at an alpha level of 0.05. Studies in various medical fields have shown similar trends in the performance of AI systems. For instance, Esteva et al. (40) demonstrated that AI could classify skin cancer with dermatologist-level accuracy, achieving a high level of agreement with expert diagnoses (40). Similarly, Mazurowski et al. (41) found that AI could significantly enhance the accuracy of radiological image analysis, aligning with our findings that AI can effectively support diagnostic processes in dentistry (41).

In dental research, Lee et al. (9) reported that a CNN-based AI system achieved high agreement values with periodontists for diagnosing periodontally compromised teeth, with kappa values of 0.828 and 0.797 for premolars and molars, respectively (9). While our AI model's kappa value of 0.445 is moderate, it demonstrates significant potential for further refinement and improvement. This analysis underscores the diagnostic capabilities of periodontist, GP, and AI in comparison to an expert. While periodontist shows the highest agreement, the AI system demonstrates promising results, potentially serving as a valuable diagnostic tool. GP, although showing lower agreement, still provide a significant level of diagnostic accuracy. These findings emphasize the importance of specialized training and the potential of AI to augment diagnostic processes in periodontal care.

The discrepancy between matrix performance and clinical accuracy can be attributed to several factors. First, differences between controlled environments and real-world variability play a significant role. In experimental settings, data is often curated and preprocessed to optimize model performance. In contrast, clinical environments present numerous uncontrolled variables, such as varying patient positioning and inconsistent imaging quality. These factors can adversely impact the performance of AI models trained under controlled conditions (42). Second, the preprocessing steps used in studies, such as noise reduction, contrast enhancement, and normalization, ensure high-quality inputs for AI models. However, in clinical practice, such preprocessing may not be consistently applied, leading to suboptimal inputs and consequently lower accuracy (8). Third, AI models often perform well on the datasets they were trained on but may struggle to generalize across diverse patient populations with different demographic characteristics, oral health conditions, and comorbidities. The training data might not fully represent the variability encountered in real clinical settings (43). Moreover, clinical diagnoses involve more than just interpreting radiographs; they require a comprehensive assessment of the patient's medical history, symptoms, and other diagnostic tests. While AI models are proficient at image analysis, they lack the ability to integrate this holistic approach, which can limit their effectiveness in real-world diagnostics (7).

The growing applications of machine learning (ML) and AI in dentistry emphasize the potential for innovative diagnostic approaches. Alharbi et al. (44) demonstrated the efficacy of machine learning models in predicting dental implant success, highlighting the role of AI in enhancing treatment outcomes through predictive analytics and tailored solutions to individual patients' needs. This aligns with our study's focus on using AI to improve diagnostic accuracy in periodontitis by leveraging data-driven insights (44). Similarly, Yadalam et al. (45) emphasized the interplay between systemic conditions, such as diabetes, dyslipidemia, and periodontitis, by predicting hub genes using interactomic approaches. Their work underscores the value of integrating systemic and periodontal diagnostics, providing a basis for holistic care strategies (45). Furthermore, Srinivasan et al. (46) successfully utilized lightweight CNN models, such as SqueezeNet, to detect smoker melanosis in gingiva, demonstrating the viability of computationally efficient models in resource-constrained settings (46). These studies collectively support the premise that AI technologies, ranging from predictive models to real-time diagnostic tools, can transform periodontal care by improving precision, efficiency, and accessibility while addressing systemic health interconnections.

Moreover, one of the critical issues highlighted is the high prevalence of periodontal disease in Thailand. The most recent survey in 2023 revealed a significant increase in periodontitis among older adults, with 48.7% of patients affected, compared to 36.3% in the previous survey. This rate far exceeds the 19% prevalence reported globally in the 2017 Global Burden of Disease Study (47, 48). These alarming statistics emphasize the urgent need for improved prevention strategies and the importance of enhancing periodontal health. The AI models developed in this study offer a promising solution by providing faster, less labor-intensive, and more accurate alternatives to current diagnostic methods. If the Ministry of Public Health recognizes the importance of this issue and supports the nationwide and global implementation of these AI models, it could significantly reduce the prevalence of periodontal disease, thereby improving public health and enhancing the quality of life.

## 5 Limitations

The current study has several limitations. First, it focused on a limited range of dental professionals, so future research should include comparisons with general practitioners and specialists. Second, the study used panoramic radiographs from a single imaging device, which may restrict the generalizability of the findings. Testing AI models on radiographs from various devices is necessary for broader applicability. Additionally, excluding certain patient demographics, such as those with rare bone morphologies or improper positioning, limits the model's scope. Future research should include a more diverse patient population to improve AI model robustness across clinical scenarios. Finally, while YOLOv8 excels in real-time object detection, it has limitations, including lower performance with small or densely packed objects, which affects fine-grained tasks such as early periodontal disease detection. It also struggles with occlusions and may sacrifice precision in complex environments, requiring fine-tuning with domain-specific data, such as dental radiographs, to enhance performance (49).

## 6 Future research directions

Future research should enhance AI in periodontal diagnostics by focusing on key areas: conducting comparative studies to validate AI against various dental expertise levels, standardizing imaging protocols to ensure consistent quality, incorporating advanced CNN architectures and algorithms for better sensitivity and accuracy, expanding datasets to include diverse demographics and clinical scenarios for greater generalizability, integrating AI with other diagnostic tools such as Cone Beam Computed Tomography (CBCT) and intraoral scanners for comprehensive assessments, and developing user-friendly, patient-centric applications for early detection and intervention.

## 7 Conclusions

This study showed that AI models can effectively identify periodontal bone loss from panoramic radiographs, offering clear advantages over classical methods. However, further research is needed to overcome existing limitations and expand AI applications in this field.

The significant economic, social, and health impacts of periodontal disease, particularly in the elderly, underscore the need for innovative diagnostic solutions. Incorporating AI technologies into periodontal care allows dental professionals to deliver faster, more precise, and comprehensive treatment, enhancing patient outcomes and reducing the burden on healthcare systems.

## Data Availability

The raw data supporting the conclusions of this article will be made available by the authors, without undue reservation.
